# Secreted nucleases reclaim extracellular DNA during biofilm development

**DOI:** 10.1038/s41522-024-00575-9

**Published:** 2024-10-07

**Authors:** Stephen M. Lander, Garth Fisher, Blake A. Everett, Peter Tran, Arthur Prindle

**Affiliations:** 1 Department of Biochemistry and Molecular Genetics, Feinberg School of Medicine, Chicago, 60611 IL USA; 2 Medical Scientist Training Program, Feinberg School of Medicine, Northwestern University, Chicago, 60611 IL USA; 3 Center for Synthetic Biology, Northwestern University, Evanston, 60208 IL USA; 4 Department of Chemical and Biological Engineering, Northwestern University, Evanston, 60208 IL USA; 5 Department of Microbiology-Immunology, Feinberg School of Medicine, Chicago, 60611 IL USA; 6 Chan Zuckerberg Biohub Chicago, Chicago, IL 60642 USA

**Keywords:** Biofilms, Microbial communities, Microbial genetics

## Abstract

DNA is the genetic code found inside all living cells and its molecular stability can also be utilized outside the cell. While extracellular DNA (eDNA) has been identified as a structural polymer in bacterial biofilms, whether it persists stably throughout development remains unclear. Here, we report that eDNA is temporarily invested in the biofilm matrix before being reclaimed later in development. Specifically, by imaging eDNA dynamics within undomesticated *Bacillus subtilis* biofilms, we found eDNA is produced during biofilm establishment before being globally degraded in a spatiotemporally coordinated pulse. We identified YhcR, a secreted Ca^2+^-dependent nuclease, as responsible for eDNA degradation in pellicle biofilms. YhcR cooperates with two other nucleases, NucA and NucB, to reclaim eDNA for its phosphate content in colony biofilms. Our results identify extracellular nucleases that are crucial for eDNA reclamation during biofilm development and we therefore propose a new role for eDNA as a dynamic metabolic reservoir.

## Introduction

DNA is commonly recognized as the genetic code found inside all living cells, but it can also be found outside the cell in bacterial, archaeal, and fungal biofilms^1–4^. In bacteria, DNA released outside the cell—known as extracellular DNA (eDNA)—facilitates biofilm formation in several species, including *Bacillus subtilis*, *Pseudomonas aeruginosa*, and *Streptococcus pneumoniae*^5–8^. The mechanism of release varies depending on the species, and can comprise active secretion, lysis, vesicular release, or a combination of these mechanisms^9,10^. In certain bacteria, the release of eDNA is regulated by the induction of natural competence through quorum sensing pathways^11^. Thus, eDNA has been linked to social behaviors associated with biofilm community development in *B. subtilis*^12^. While the role of eDNA in bacterial biofilms differs depending on the species, eDNA is generally considered a key component of biofilm formation.

The chemical properties of eDNA can contribute to biofilm fitness by altering the cellular microenvironment. Specifically, the negative charge of eDNA attracts and sequesters cations such as Ca^2+^, Mn^2+^, Mg^2+^ and Zn^2+^, which can indirectly increase antimicrobial resistance in biofilms^13^. In *B. subtilis*, lower Mg^2+^ concentrations are associated with decreased survival of cells exposed to antibiotics^14^. Similarly, loss of Mg^2+^ in *P. aeruginosa* induces changes to the lipopolysaccharides that mask the negative charge of the bacteria and reduces the efficacy of antimicrobial peptides and certain cationic antibiotics^15,16^. In some cases, eDNA is also thought to directly bind and inhibit the diffusion of cationic peptides or antibiotics in biofilms^17–19^. Similarly, eDNA could influence the diffusion of other cationic signals involved in biofilm development, such as K^+^ or H^+^^20–22^. Thus, the chemical properties of eDNA can contribute to the established properties of biofilms, such as antibiotic resistance.

The unique biophysical properties of eDNA can also play a role in biofilm formation. eDNA facilitates the adhesion of bacterial cells to surfaces by reducing the radius of the contact region between the bacterial cell and the surface, interacting with pores of certain surfaces with nano-scale roughness, and increasing the hydrophobicity of the cell for better adherence^23–25^. Once a biofilm has formed, eDNA can further contribute to its stability. For example, eDNA alters the structure of exopolysaccharide in *B. subtilis* biofilms and increases the biomass of its pellicles^26^. Furthermore, the addition of eDNA was shown to increase the size of aggregates in *B. subtilis* and other species^27,28^. Thus, eDNA can contribute to biofilm establishment and the formation of mature components of the biofilm extracellular matrix, such as exopolysaccharides. Conversely, DNase has been shown to break up aggregates in multiple species, prompting the exploration of DNases as anti-biofilm treatments^5,29–31^. However, the efficacy of these treatments can depend on timing, with studies revealing that immature biofilms are more susceptible to DNase than mature biofilms^5,26,32^. Together, these studies suggest that the role of eDNA in biofilms may be transient, highlighting the need for a spatiotemporal analysis of eDNA within biofilms.

Considering that eDNA is a metabolically demanding molecule to synthesize, we hypothesize that eDNA acts as a metabolic reservoir that the biofilm can reclaim when nutrient availability diminishes later in development^20,33–37^. Here, we report the discovery of spatiotemporal eDNA dynamics during *B. subtilis* biofilm development, the identification of the nucleases responsible for coordinating these dynamics, and the demonstration of a new role for eDNA as an extracellular phosphate reservoir.

## Methods

### Growth conditions

Bacteria were grown in lysogeny broth (LB) rich media and seeded into MSgg-defined minimal media. Biofilms were grown in standard MSgg, which contains 100 mM MOPS, 5 mM potassium-phosphate buffer (pH 7), 2 mM MgCl_2_, 700 µM CaCl_2_, 50 µM MnCl_2_, 100 µM FeCl_3_, 1 µM ZnCl_2_, 2 µM thiamine HCl, 0.5% (v/v) glycerol, and 0.5% (w/v) monosodium glutamate. All MSgg liquid was filtered using a Steriflip 0.22 µM filter (Millipore SCGP00525) before using for pellicle and DNase assays. Solid MSgg (1.5% agar) was autoclaved, and the glutamate and dyes were added after cooling to 55°C. Strains were grown to consistent OD600 0.8-1.3 in LB for all strains, spun down, washed in 1x PBS, and resuspended in 1x PBS. 2 µL of cell culture was then seeded into 198 µL MSgg in a 96-well microplate for pellicle biofilms (Corning 3904) or 0.5 µL into a well with 0.6-1.0 mL solid MSgg agar in a 24-well plate for colony biofilms (Corning 3526). To measure biofilm eDNA, 1 µM TOTO-1 was added to the media. To measure the biofilm matrix, 20 µg/mL of Congo Red was used. Growth experiments with eDNA as the sole phosphate source were conducted with the potassium phosphate buffer replaced with equimolar KCl and 0.5 mg/mL UltraPure Salmon Sperm DNA Solution (Invitrogen 1563201). Biofilms were grown at 30°C without shaking.

### Optical density, fluorescence, and pellicle biofilm measurements

TOTO fluorescence for pellicle biofilms in all our studies were measured using a TECAN Infinite MPLEX plate reader with excitation/emission wavelength set to 488/540 nm and gain set to 150. Pellicle biofilm assays were conducted without shaking at 30°C with measurements being taken every 15 minutes. Safranin staining was measured at OD530.

### DNA cloning

Genetic complement with native promoters were amplified from the genome of the wild-type NCIB 3610 strain with 500 bp of the native promoters and added to the integration vector ECE174 (https://bgsc.org/search.php?Search=ece174) with chloramphenicol resistance. Primers used for amplifying *yhcR* gene and *yhcR* native promoter for the Gibson construct were GATAAGCTGTCAAACATGAGGCATAGAAGCTTGTGCTTTAATCGC and CCGGCGCTCAGGATCCTAGATCACGTTCTGGAGGCGC. Primers used for amplifying the vector backbone were GGAGCGCCTCCAGAACGTGATCTAGGATCCTGAGCGCCG and TAAAGCACAAGCTTCTATGCCTCATGTTTGACAGCTTATCATCGG. All plasmid assembly was performed using Gibson Assembly using the Gibson Assembly Master Mix (NEB). The assembled plasmid was transformed into NCIB 3610 using a natural competence protocol previously described and plated on LB agar with appropriate selection^38^.

### DNase activity assay

Pellicle biofilms were grown in MSgg in 96-well plates. For each time point, 24 wells were pooled, centrifuged at 800rcf for 10 minutes, and then 0.22 µm filtered (Millipore GPWP04700) to yield cell-free filtrate. This filtrate then had 1 µM TOTO-1 and 10 ng/µL salmon sperm DNA added, and the TOTO-1 signal of 200uL wells was measured over time in the plate reader. The negative slope of the linear section of this decay curve was analyzed as the amount of relative DNase activity.

### Nuclease co-factor complementation

The DNase activity assay was applied to extracellular samples that had all free metal ions chelated by the addition of 10 mM EDTA. The ion of interest was then added at 100 times the concentration present in MSgg to overwhelm the chelation, leaving it as the only free metal cation.

### Proteomics

Extracellular filtrate was prepared in the same manner as the secreted nuclease assay at four time points during pellicle biofilm development ranging from approximately 20 to 100 hours. The resulting extracellular samples were concentrated using Amicon Ultra-15 centrifugal filters with a 10kda molecular cut off (Amicon UFC901008) and washed with 10 mL of 10 mM ammonium bicarbonate. Secretome samples were submitted to the Northwestern proteomics core, where they underwent an acetone/TCA protein precipitation to generate pellets. Protein digestion was performed by trypsin addition (Promega). Sample proteomes were measured by LC-MS/MS using a DionexUltiMate 3000 Rapid Separation LC system and a linear ion trap-Orbitrap hybrid Elite mass spectrometer (Thermo Fisher Scientific Inc). MS/MS spectra were matched against the UniProt reference proteome for B. subtilis 168 (UP000001570). At the peptide level a false discovery rate (FDR) cutoff of 1% was applied. Only proteins with more than one peptide were considered for further study. Identified peptides and proteins were analyzed for abundance using Scaffold software (version 5.0, Proteome Software Inc., Portland, OR). Putative nucleases were identified by searching Subtiwiki functional annotations.

### RNA isolation

*B. subtilis* NCIB 3610 pellicle biofilms were grown for 82 h in MSgg media. 24 wells were harvested by mixing pellicles and supernatant into an equal volume of −80°C pre-chilled methanol, yielding a 50% methanol solution. The biofilm was then pelleted by centrifugation at 4°C, the supernatant was removed, and cells were resuspended into 1 mL of pre-chilled 50% methanol solution. The biofilm was then pelleted again, aspirated to remove supernatant and flash frozen with liquid nitrogen before being stored overnight at −80°C. RNA was then isolated using the QIAGEN Rneasy kit according to the manufacturer’s instructions with lysis being completed by 30 s of bead-beating using Lysis Matrix B tubes in the Omni Bead Ruptor Elite machine.

### RNA-sequencing

RNA quality was checked using Bioanalyzer (Agilent) prior to RNA-seq library preparation. RNA samples with an RNA integrity number >8 were used for library preparation, which was constructed from 35 ng of RNA with the Illumina Stranded Total RNA Prep, Ligation with Ribo-Zero Plus kit (Illumina). RNA Sequencing was then performed on NextSeq 500 sequencer and analyzed as previously described. The quality of reads, in FASTQ format, was evaluated using FastQC. Reads were trimmed to remove Illumina adapters from the 3’ ends using cutadapt^39^. Trimmed reads were aligned to the *B. subtilis* genome strain 3610 NCIB CP020102.1 and plasmid NCIB CP020103.1 using STAR^40^. Read counts for each gene were calculated using htseq-count in conjunction with a gene annotation file for the reference genomes obtained from NCBI. Normalization and differential expression were calculated using DESeq2 that employs the Wald test^41,42^. The cutoff for determining significantly differentially expressed genes was an FDR-adjusted *p*-value less than 0.05 using the Benjamini-Hochberg method.

### Microscopy

Biofilm growth was recorded using 10x phase contrast microscopy and eDNA was measured using fluorescence microscopy. Phase and fluorescent images were taken with a Nikon Ti2 and enclosed stage within an incubator set up to maintain a plate temperature of 30°C. The phase and fluorescent images were taken using the median of a 4 × 4 bin on capture. In order to image the entire biofilm and well, the 10x objective was used with ND acquisition in NIS-elements software with the built-in stitching function. For 12-well plates, an 8 by 8 image stitch was performed with built in settings within ND acquisition. Biofilm growth and eDNA dynamics were measured using images, which were taken every hour. Whenever fluorescence images were recorded, we used the minimum exposure time that still provided a good signal-to-noise ratio; phase images were taken with an exposure of 3.3 ms and an appropriate aperture setting to prevent overflow. eDNA was measured by incorporating 1 µM TOTO-1 into the agar media at set up and with an excitation/emission of 508/560 and an exposure of 50 ms. Consumption of eDNA was tracked with 1 µM TOTO-1 but exposure was lowered to 12 ms to avoid saturation. Biofilm matrix was measured by incorporating 20 µg/mL of Congo Red Dye into the agar media and with an excitation/emission of 470/614 nm and exposure of 20 ms. Laser power for eDNA and Congo Red dyes was set at 100%. FITC emission filter cube was used for eDNA and Congo Red images. Images for the timelapse and traces were taken every hour.

### Image analysis

Fiji/ImageJ (National Institutes of Health) was used for image analysis. To measure biofilm fluorescence, we identified the biofilm area first using phase and creating custom regions of interests (ROIs) that outlined the biofilm for each frame. We then used the same ROIs on the relevant fluorescent channel of the same experimental run to measure average fluorescent reporter signal over time.

### Statistical analyses

Statistical tests were calculated in MATLAB and python. For comparisons between two independent groups, a Student’s T-test was used. Significance was accepted at *p* < 0.05. The details of the statistical tests carried out are indicated in respective figure legends.

## Results

### Spatiotemporal dynamics of eDNA during biofilm development

**Fig. 1:**
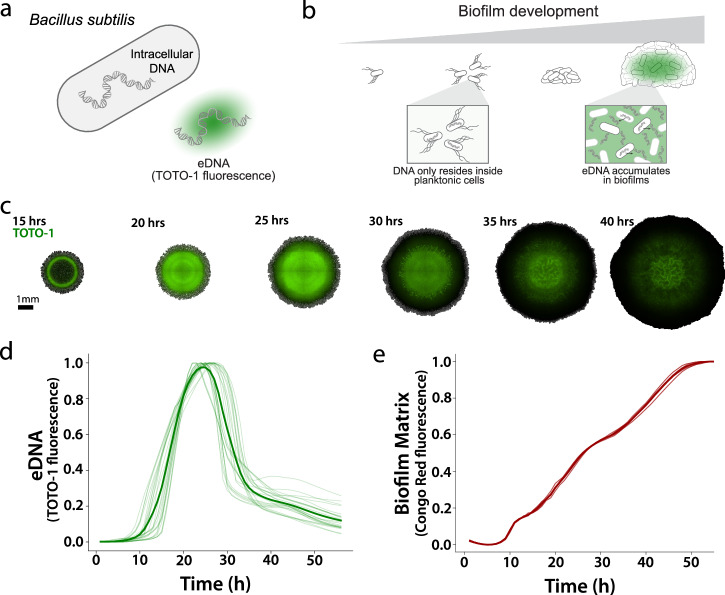
Spatiotemporal dynamics of eDNA during biofilm development. **a** Cell-impermeant extracellular dye TOTO-1 binds eDNA and fluoresces. **b** Stages of biofilm development with eDNA accumulating in the biofilm matrix. **c** Representative merged phase and TOTO-1 fluorescence images of a developing *B. subtilis* NCIB 3610 wildtype colony biofilm on solid MSgg medium. Background was cropped using the edge of the biofilm. **d** eDNA dynamics of 26 wild-type colony biofilms on solid MSgg medium as measured by TOTO-1 fluorescence. The peaks have been normalized in amplitude and shifted in time to align them for comparison. The mean fluorescence is shown in bold. **e** Matrix accumulation over development of wildtype colony biofilms on solid MSgg medium measured by Congo Red fluorescence (*n* = 4). The mean fluorescence is shown in bold.

We established an experimental system capable of tracking extracellular DNA dynamics in undomesticated *B. subtilis* NCIB 3610 biofilms. NCIB 3610 is an established eDNA producer that produces approximately 40 times more eDNA than nonbiofilm-forming laboratory-adapted strains, *B. subtilis* 168^12,43^. To visualize eDNA in biofilms we utilized TOTO-1, a cell-impermeable DNA dye that has been previously used to study eDNA (Fig. 1a)^26,44^. We confirmed that TOTO-1 fluorescence scales linearly with eDNA concentration in standard MSgg minimal media (Supplementary Figure 1). We could then grow biofilms on solid MSgg agar with TOTO-1 dye to track eDNA by timelapse fluorescence microscopy (Fig. 1b). This experimental system thus enables spatiotemporal tracking of eDNA during *B. subtilis* biofilm development.

Using this experimental system, we found that *B. subtilis* displays striking eDNA dynamics during biofilm development (Supplementary Movie 1). Specifically, eDNA is produced throughout the entire biofilm before being removed, yielding a spatiotemporally coordinated pulse (Fig. 1c). We observed this pulsatile eDNA behavior despite continued biofilm development as measured by the biofilm matrix dye Congo Red which binds protein fibrils (Fig. 1d). The appearance and apparent degradation of eDNA therefore occurred alongside active production of TasA, an established proteinaceous extracellular matrix component in *B. subtilis*^45^. Thus, eDNA dynamics occurred during normal biofilm development. The biofilm diameter at the eDNA peak is centered at 8 mm ending at a maximum of 12 mm (*n* = 26 biofilms, Supplementary Fig. 2). We verified that observed eDNA degradation is not due to TOTO-1 dye photobleaching (Supplementary Fig. 3). These results suggest that eDNA is transiently produced and then degraded during biofilm development.

### An extracellular Ca^2+^-dependent nuclease is responsible for eDNA dynamics

Next, we explored multiple potential mechanisms of eDNA depletion, including extracellular nucleases and competence uptake. We began with NucA and NucB, which are extracellular non-specific manganese-stimulated endonucleases that can cleave ssDNA as well as dsDNA^46,47^. NucA is an integral membrane protein regulated by the induction of natural competence that catalyzes dsDNA cleavage for transformation^48^. NucB is a secreted sporulation-specific extracellular nuclease and has been shown to have biofilm-dispersing properties in *Bacillus lichenformis*^49^. While *ΔnucA* and *ΔnucB* mutant biofilms showed slight increases in the eDNA peak and duration, the eDNA pulse remained largely intact (Supplementary Fig. 4). We further investigated the essential components of the natural competence uptake machinery, but they were similarly unnecessary for eDNA degradation (Supplementary Fig. 4). Recent studies have shown competence may be inversely regulated in relation to biofilm development^50^. Thus, we suspected that an unidentified extracellular nuclease is responsible for eDNA degradation.

**Fig. 2:**
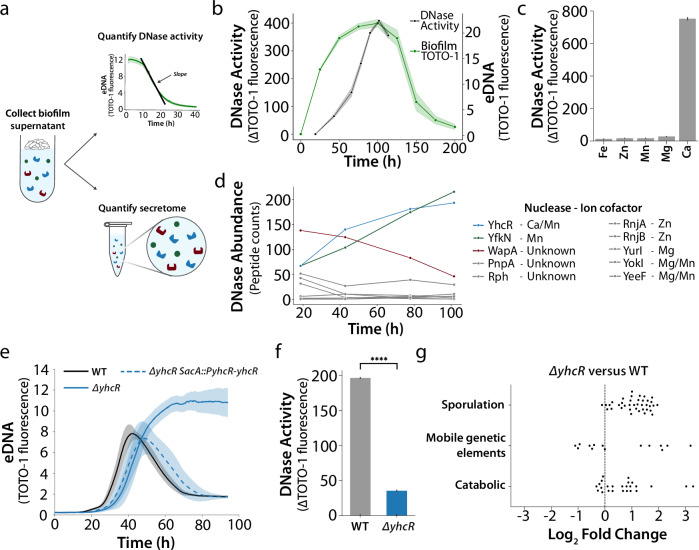
YhcR is responsible for degrading eDNA in pellicle biofilms. **a** For the secretomics experiment pellicle biofilms were grown at the air-liquid interface of MSgg medium in 96-well plates, pooled, centrifuged, and filtered to select all secreted proteins. DNase activity of the filtered supernatant was estimated as the negative slope of the TOTO-1 fluorescence following DNA addition. The secretome of these timepoints was also quantified via proteomics. **b** eDNA dynamics of wildtype pellicle biofilms grown in liquid MSgg. Secreted DNase activity of pellicle biofilms showing an increase throughout biofilm development (*n* = 3, 24 pooled pellicles). Traces are the mean and shaded area is the standard deviation. **c** DNase assay of pellicle supernatant chelated of all metal ions by addition of 10 mM EDTA followed by chemical complementation of each ion (*n* = 3). Bars display the mean and error bars represent the standard deviation. **d** Relative abundance of the 10 nucleases present in the secretome over time, where only YhcR and YfkN increase in abundance throughout biofilm development (*n* = 1, 24 pooled pellicles). **e** eDNA dynamic of *ΔyhcR* pellicle biofilms over development compared to a wildtype control and a genetic complement for YhcR with the native *yhcR* promoter (*n* = 12). Traces are the mean and shaded area is the standard deviation. **f** Secreted DNase activity of *ΔyhcR* pellicle biofilms compared to a wildtype control at 48 hours into development (*n* = 3). Bars display the mean and error bars represent the standard deviation. Statistical significance was calculated using a Student’s t-test with *p* = 2.02E-13. **g** Log_2_ fold-enrichment of selected functional category terms in the *ΔyhcR* mutant as a swarm plot. Functional Enrichment Analysis (FEA) was performed using Subtiwiki functional categories.

To interrogate extracellular nuclease activity, we cultured pellicle (air-liquid interface) biofilms to enable direct sampling and analysis of the extracellular compartment, known as the secretome (Fig. 2a). After harvesting the pellicle biofilm supernatant, we first quantified nuclease activity using the negative slope of TOTO-1 signal following exogenous eDNA addition. We measured nuclease activity that increased throughout biofilm development and reached a maximum at the onset of biofilm eDNA degradation (Fig. 2b). Extracellular nuclease activity was diminished by heating and proteinase K, suggesting that the activity was caused by a protein (Supplementary Fig. 5). Since nucleases use specific metal ion cofactors, we systemically repeated nuclease activity assays in the presence of single cations. We observed a loss of nuclease activity that could only be rescued by Ca^2+^ (Fig. 2c). Together, these results suggest that an extracellular Ca^2+^-dependent nuclease is responsible for eDNA dynamics.

### YhcR is responsible for degrading eDNA in pellicle biofilms

**Fig. 3:**
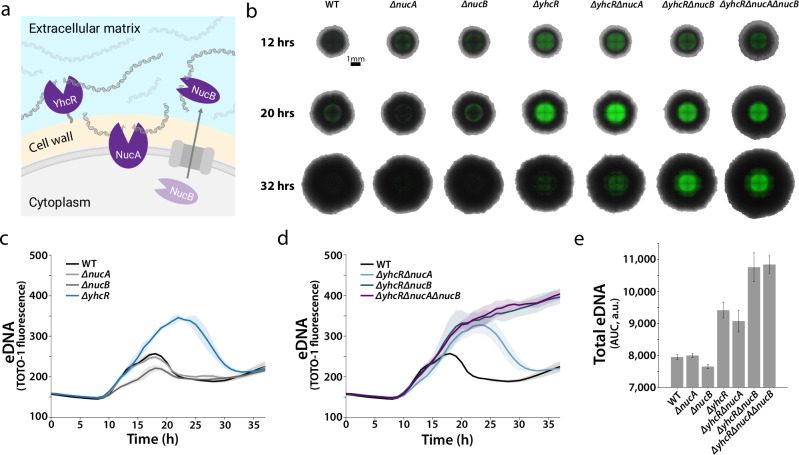
NucA and NucB cooperate with YhcR to degrade eDNA in colony biofilms. **a** Extracellular location of the three identified nucleases—YhcR, NucA, and NucB—according to existing literature^46,47,52–54^. **b** Representative merged Phase and TOTO-1 fluorescence images of *B. subtilis* NCIB 3610 biofilm nuclease mutants on solid MSgg medium. Background was cropped using the edge of the biofilm. **c** and **d** eDNA dynamics of wildtype and mutant biofilms on solid MSgg medium. Traces are mean fluorescence of biofilms (*n* = 3), and shaded area is standard deviation for all biofilms grown in a single head-to-head experiment. **e** Relative total eDNA estimated by taking the area under the TOTO-1 fluorescence curves in panels c and d (*n* = 3). Bars display the mean and error bars represent the standard deviation.

To identify the nuclease responsible for eDNA degradation, we submitted the secreted proteins for analysis by mass spectrometry. We validated that our workflow predominantly detects secreted proteins identified in previous studies of the *B. subtilis* secretome as annotated in the Subtiwiki knowledgebase (Supplementary Fig. 6)^51^. Amongst the 350 proteins detected, our proteomics workflow identified 10 putative nucleases secreted during biofilm development that could be responsible for eDNA degradation (Fig. 2d). By comparing the previously determined nuclease activity profile with the nuclease abundances (Fig. 2b), we narrowed down on two proteins—YhcR and YfkN—whose abundance appeared to track with nuclease activity (Fig. 2d). Of these, YhcR is also the only identified nuclease reported to utilize Ca^2+^ amongst those detected^51,52^. We therefore suspected YhcR to be the secreted nuclease responsible for eDNA degradation during biofilm development (Fig. 3a).

YhcR is a non-specific endonuclease that is secreted via the Sec protein translocation machinery and anchored to the cell wall by a sortase in the same operon, YhcS^53,54^. We generated *ΔyhcR* mutant biofilms and repeated the same extracellular nuclease activity and eDNA measurements as before. As expected, when we tracked TOTO-1 fluorescence in *ΔyhcR* mutant pellicle biofilms we observed a complete loss of eDNA degradation (Fig. 2e). The requirement of YhcR for eDNA degradation was confirmed by genetic complementation (Fig. 2e). We also observed that *ΔyhcR* mutant biofilms are deficient in extracellular nuclease activity (*p* = 2.02E-13, Student’s t-test, Fig. 2f). In both cases, these results recapitulate the loss of eDNA degradation and nuclease activity in the absence of Ca^2+^ (Supplementary Figure 7). Additionally, *ΔyfkN* mutant biofilms did not show a significant difference in eDNA degradation when compared with wildtype (WT) (Supplementary Fig. 8). Together, these results confirm that YhcR is responsible for degrading eDNA in pellicle biofilms.

To determine the functional impact of eDNA degradation in biofilms, we performed RNA-seq on WT and *ΔyhcR* mutant biofilms. We identified differentially expressed genes and performed functional enrichment analysis to identify pathways dependent on YhcR activity. We found that *ΔyhcR* mutant biofilms exhibit enrichment of sporulation pathways, which are activated when nutrients become limiting or during altered biofilm development (Fig. 2g). We also found enrichment of the primary mobile genetic element identified is the ICEBs1 integrative and conjugative element (Fig. 2g)^55^. Furthermore, we saw increased carbon and polysaccharide catabolic processes (Fig. 2g). Taken together, these results suggest increased starvation in *ΔyhcR* mutant biofilms compared to wildtype. We therefore suspected that YhcR could be involved in nutrient acquisition during biofilm development.

### YhcR cooperates with NucA and NucB to degrade eDNA in colony biofilms

To test this hypothesis, we grew colony biofilms on solid agar media to analyze spatiotemporal eDNA dynamics in the identified nuclease mutants. While the peak of the eDNA pulse was increased in *ΔyhcR* colony biofilms, we ultimately observed comparable eDNA degradation to wildtype (Fig. 3b, c). This is likely due to differences observed between pellicle biofilms grown at the air-liquid interface and colony biofilms gown on solid agar medium. These differences are likely due to morphologic, transcriptional, and post-translational changes^56,57^. However, two double nuclease mutants, *ΔyhcRΔnucA* and *ΔyhcRΔnucB*, further decreased eDNA degradation (Fig. 3b, d). Furthermore, the triple mutant—*ΔyhcRΔnucAΔnucB—*exhibited a complete lack of eDNA degradation (Fig. 3b, d). We quantified the levels of eDNA for all strains using the area under the curve to approximate eDNA abundance (Fig. 3e). These results suggest that NucA and NucB cooperate with YhcR to degrade eDNA during colony biofilm maturation.

### YhcR, NucA, and NucB cooperate to reclaim eDNA for its phosphate content in biofilms

**Fig. 4:**
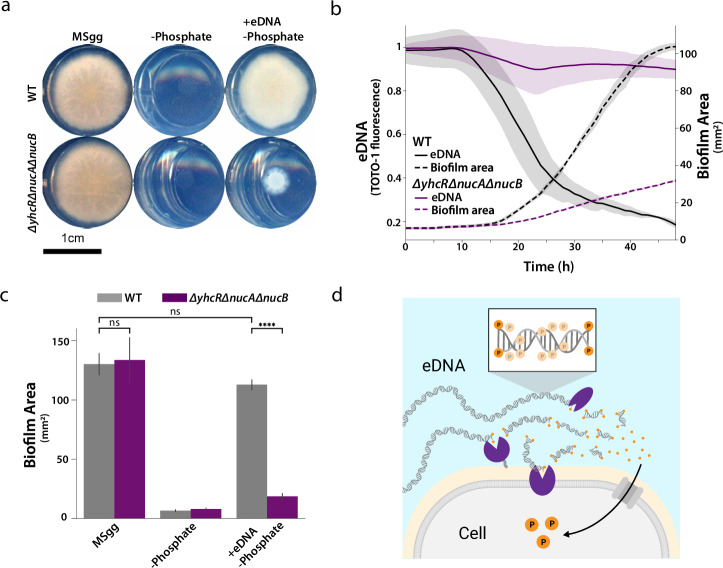
Three nucleases reclaim eDNA in colony biofilms to increase biofilm fitness. **a** Representative scanned colony biofilms grown on solid MSgg for 66 hours with phosphate, without phosphate, or with phosphate source replaced by exogenous eDNA. Background was cropped from images using edge of the wells. **b** Colony biofilms grown with exogenous eDNA as the sole phosphate source on solid MSgg medium (*n* = 3). eDNA degradation and consumption was tracked in situ by TOTO-1 fluorescence microscopy. Traces are the mean fluorescence in the biofilm and the shaded area is the standard deviation. **c** Area of scanned biofilms grown on MSgg for 66 hours with phosphate, without phosphate, or MSgg with phosphate replaced with exogenous eDNA (*n* = 3). Bars display the mean and error bars represent the standard deviation. Statistical significance was calculated using a Student’s t-test: *p* = 0.78 for wildtype and *ΔyhcRΔnucAΔnucB* grown in MSgg, *p* = 0.09 for wildtype grown in MSgg and wildtype grown in MSgg +eDNA -Phosphate, and *p* = 5.67E-5 for wildtype and *ΔyhcRΔnucAΔnucB* grown in MSgg +eDNA -Phosphate. **d** Three nucleases—YhcR, NucB, and NucA—reclaim eDNA for its phosphate content.

Since producing eDNA requires a significant investment of resources, we wondered whether eDNA could act as an extracellular nutrient reservoir for the biofilm^36,37^. Specifically, YhcR expression is increased under phosphate limitation and NucB is similarly expressed under phosphate-limited sporulation conditions^58,59^. Thus, we hypothesized that biofilms could reclaim phosphate from eDNA via the identified nucleases during biofilm maturation. To test this, we grew *B. subtilis* colony biofilms on exogenous eDNA as a sole phosphate source to mimic phosphate limitation during biofilm maturation. We first confirmed that wildtype biofilms are capable of reclaiming phosphate from exogenous eDNA to sustain growth (Fig. 4a). In contrast, the triple mutant colony biofilms grew markedly worse in the absence of phosphate (Fig. 4a). These results suggest that YhcR, NucA, and NucB are crucial for reclaiming exogenous eDNA to sustain biofilm growth in the absence of phosphate.

To directly demonstrate eDNA reclamation in biofilms, we tracked biofilm growth and exogenous eDNA degradation simultaneously. As expected, we saw that wildtype biofilms begin growing shortly after the onset of exogenous eDNA degradation, consistent with reclaiming eDNA for its phosphate content (Fig. 4b). In contrast, triple mutant biofilms grew markedly slower and failed to reclaim exogenous eDNA for its phosphate content (Fig. 4b). As such, the triple mutant fails to form robust biofilms when grown on exogenous eDNA as a phosphate source (*p* = 5.67E-5, Student’s t-test), while wildtype biofilms grow comparably to those formed in standard MSgg (Fig. 4c). This result was consistent with a range of exogenous eDNA concentrations provided to WT and the triple mutant (Supplementary Figure 9a). Furthermore, excess phosphate did not alter the growth rates of WT or the triple mutant (Supplementary Fig. 9b). These experiments establish that exogenous eDNA can be reclaimed by the cooperative activity of YhcR, NucA, and NucB to sustain growth in the absence of free phosphate (Fig. 4d). Thus, these results reveal a new role for eDNA as a dynamic metabolic reservoir capable of providing phosphate for biofilm maturation.

## Discussion

The genes inside of all living cells are encoded in the sequence of DNA, a polymer whose unique structural stability can also be utilized outside the cell^9,10,60^. By imaging eDNA dynamics within undomesticated *B. subtilis* NCIB 3610 biofilms we found that eDNA is temporarily invested in the biofilm matrix before being later metabolized for cell growth. Our results demonstrate a crucial eDNA reclamation role for a secreted nuclease, YhcR, during biofilm development. While we did not address the mechanism of eDNA production, prior work has identified both active release from living cells as well as autolysis or programmed cell death as possible mechanisms. In some cases, these processes may be regulated by quorum sensing pathways including surfactin that links the expression of the competence machinery to the release of eDNA^61^. Thus, future work might explore the transcriptional regulation of YhcR expression, which is not currently known to be associated with quorum sensing processes or other biofilm regulatory pathways.

DNA is an energy intensive molecule to make that requires key essential nutrients including carbon, nitrogen, and phosphate^36,37^. Based on the molecular structure of DNA, phosphate is probably the least enzymatically complex nutrient to reclaim. Since YhcR, NucA, and NucB are all non-specific endonucleases, they can cleave the eDNA backbone to expose phosphate ends. YfkN is an extracellular phosphodiesterase that is induced during phosphate limitation and reportedly co-localizes with YhcR^53,59^. It is therefore possible that YhcR and YfkN coordinate to efficiently harvest free phosphates from eDNA. Thus, eDNA could serve as a phosphate reservoir to complement known bacterial phosphate reservoirs such as the cell wall^62^. Beyond self-produced eDNA, neighboring species undergoing programed cell death or lysis during microbial warfare could also release eDNA into the environment^9^. Harvesting of the free nucleobases may also promote biofilm formation in certain bacteria^63^. Thus, the identified nucleases may not only reclaim self-produced eDNA but could also aid the biofilm in nutrient harvesting from nearby species.

In addition to mitigating nutrient depletion, there are other possible functional roles for reclaiming eDNA in biofilms. eDNA may act as a transient scaffold that facilitates formation of mature biofilm extracellular matrix components, such as anchoring proteins^26^. Indeed, such a scaffolding role has been recently reported in a gut symbiont^64^. Separately, it has also been suggested that nucleases produced in biofilms may be associated with dispersal back to the planktonic lifestyle^47,49,65^. eDNA could represent a provisional commitment to the biofilm lifestyle that can be reversed upon degradation. Additionally, our results could explain why early biofilms are sensitive to DNase while mature biofilms are often not, as well as why DNase treatment has generally not been successful as an antibiofilm method. Based on the role of eDNA in antibiotic resistance, it is also possible that Ca^2+^ or Mn^2+^ modulators could potentially be repurposed as antibiotic adjuvants to prevent eDNA reclamation. Furthermore, calcium was shown to stabilize the *B. subtilis* biofilm matrix and prevent dispersion—promoting robust biofilm development^66,67^. Given the key influence of calcium on biofilm stabilization and eDNA dynamics, it will be interesting to explore how manipulating eDNA and calcium levels may overcome antimicrobial resistance properties in biofilms.

Lastly, recent studies have reported that *B. subtilis* engages in cell-to-cell horizontal gene transfer (HGT)^9,12,68,69^. HGT is thought to occur at a higher rate within biofilms, and eDNA is a potential source of genetic transfer between neighboring cells^70,71^. It is then intriguing to think that the eDNA dynamics observed here may temporally coordinate HGT during biofilm development, providing a shared transient genetic reservoir for the community. While natural competence is a known property of *B. subtilis*, competence has been typically understood as a stochastic behavior associated with single cells rather than a coordinated process. Competence may play an additional role in the utilization of eDNA, where YhcR, NucA, and NucB could generate eDNA fragments to be transformed via natural competence^46^. Furthermore, subpopulations within biofilms are known to be differentially regulated such competence cells are prohibited from becoming matrix producers, so there may be a spatial organization to HGT within distinct subpopulations found in the biofilms^50^. Therefore, eDNA degradation during biofilm maturation could potentially facilitate HGT in addition to nutrient reclamation.

## Supplementary information

Supplemental Information

Supplementary Movie 1

## Data Availability

The RNA sequencing dataset generated during the current study are available in the NCBI BioProjects repository, ID: 1086562, to be released upon publication. Other datasets used and/or analyzed during the current study available from the corresponding author on reasonable request.
