# Author Correction: Secreted nucleases reclaim extracellular DNA during biofilm development

**DOI:** 10.1038/s41522-024-00595-5

**Published:** 2024-10-31

**Authors:** Stephen M. Lander, Garth Fisher, Blake A. Everett, Peter Tran, Arthur Prindle

**Affiliations:** 1 Department of Biochemistry and Molecular Genetics, Feinberg School of Medicine, Chicago, 60611 IL USA; 2 Medical Scientist Training Program, Feinberg School of Medicine, Northwestern University, Chicago, 60611 IL USA; 3 Center for Synthetic Biology, Northwestern University, Evanston, 60208 IL USA; 4 Department of Chemical and Biological Engineering, Northwestern University, Evanston, 60208 IL USA; 5 Department of Microbiology-Immunology, Feinberg School of Medicine, Chicago, 60611 IL USA; 6 Chan Zuckerberg Biohub Chicago, Chicago, IL 60642 USA

**Keywords:** Biofilms, Microbial communities, Microbial genetics

Correction to: *npj Biofilms and Microbiomes* 10.1038/s41522-024-00575-9, published online 07 October 2024

In this article the grant number “Simons Foundation MP-TMPS-00005320 and NSF DMS-2235451” relating to National Institute for Theory and Mathematics in Biology was omitted. The original article has been corrected.

