# Supplementary material for: Secreted nucleases reclaim extracellular DNA during biofilm development
Source: NPJ Biofilms Microbiomes. 2024 Oct 7;10:103. doi: 10.1038/s41522-024-00575-9 (PMC11458576; doi:10.1038/s41522-024-00575-9)
Supplement: Supplementary file 1 — Supplemental Information [file 41522_2024_575_MOESM1_ESM.pdf]

Supplemental Information for

Secreted nucleases reclaim extracellular DNA during biofilm development

Stephen M. Lander*, Garth Fisher*, Blake A. Everett, Peter Tran, Arthur Prindle
*These authors contributed equally to this work

Corresponding and lead author, Arthur Prindle
Email: arthur.prindle@northwestern.edu

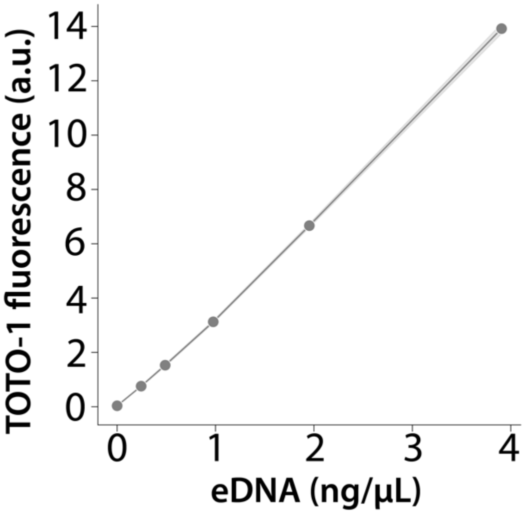

Supplementary Figure 1. TOTO-1 fluorescent dye scales with DNA concentrations. DNA concentrations were prepared with UltraPure Salmon Sperm DNA and mixed with 1 µM TOTO-1 in a 96-well plate. Fluorescence was read using the Tecan Plate reader with the settings provided in the materials section. Dots represent average measurements for n=3 and standard deviations is shown with shaded area.

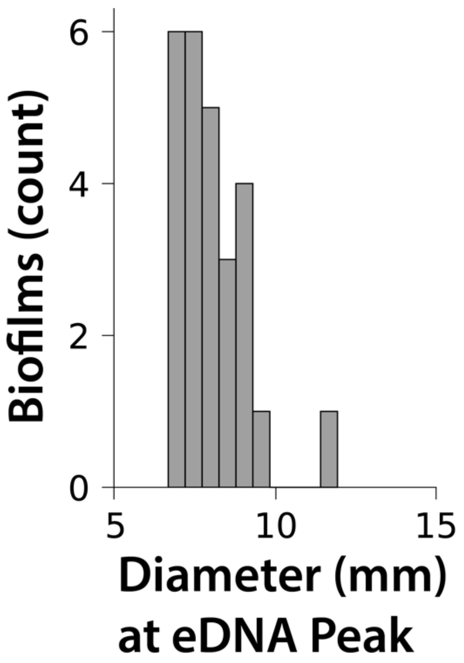

Supplementary Figure 2. Distribution of the diameter of the biofilms at the time of peak eDNA concentration. Diameter at eDNA peak was measured for all biofilms included in analysis for Fig. 1d (n=26).

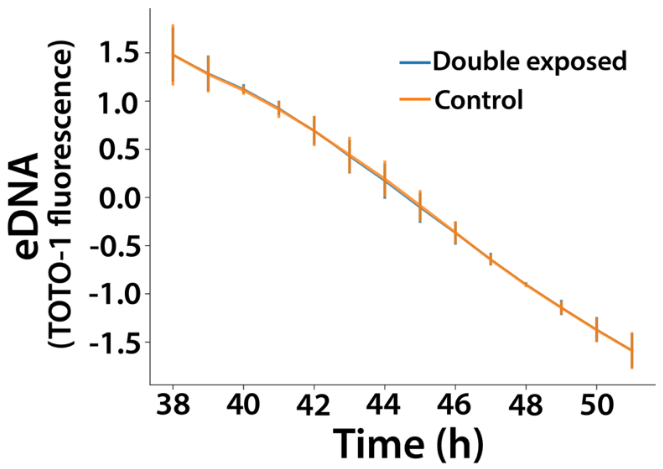

Supplementary Figure 3. TOTO-1 signal degradation is not due to photobleaching. Half of a single biofilms was imaged with a the normal 50ms of exposure for TOTO-1, and half of the same biofilm was imaged with double the exposure, 100ms. The measurements for TOTO-1 were analyzed and plotted for each side of the biofilm.

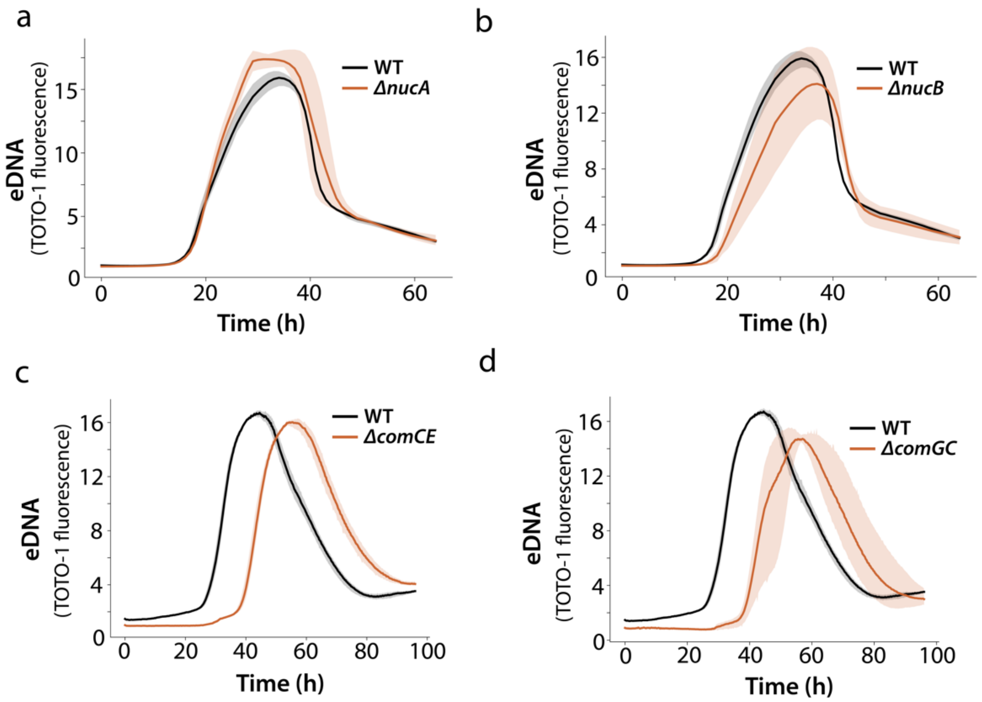

Supplementary Figure 4. NucA, NucB, and competence uptake machinery mutants still have eDNA pulse including eDNA degradation. eDNA dynamics of wildtype and mutant biofilms on solid MSgg medium a) *ΔnucA*, b) *ΔnucB*, c) *ΔcomEC*, d) *ΔcomGC*. Traces are mean fluorescence of biofilms (n=3), and shaded area is standard deviation for all biofilms. Each mutant biofilm was grown in a separate experiment, and each experiment had a direct wildtype control for comparison.

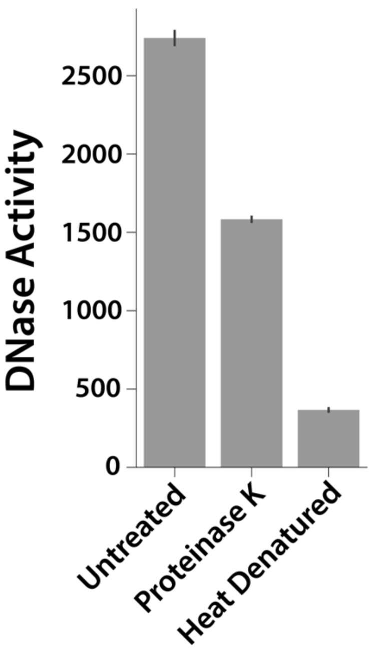

Supplementary Figure 5. Heat denaturing and proteinase K reduce DNase activity in pellicle biofilm supernatant. DNase activity of untreated, proteinase K treated, and heat denatured supernatant (n=3). Proteinase K sample was treated for 10 minutes with final concentration of 2 mg/mL. For heat denaturing, sample was placed at 95°C for 10 minutes. Error bars represent the standard deviation.

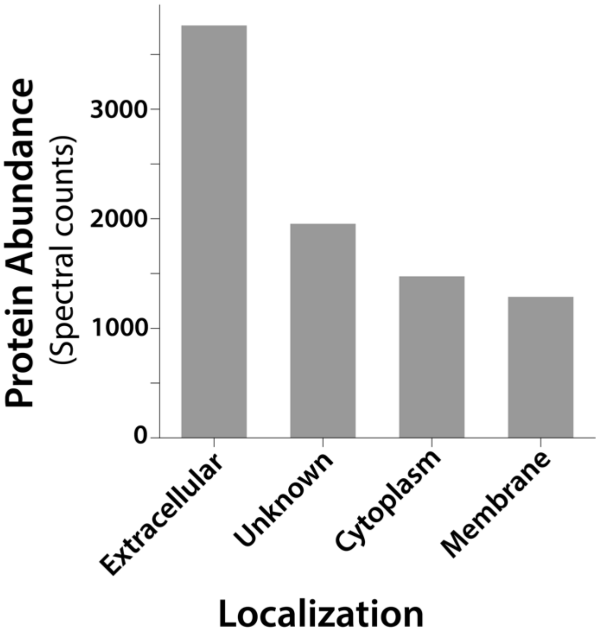

Supplementary Figure 6. Secretomics workflow correlated with previously identified secreted proteins. Localization of the detected proteins (350 total unique proteins) in the proteomics samples was determined using the literature and subtiwiki localization and grouped into extracellular, unknown, cytoplasmic, and membrane localization categories[38].

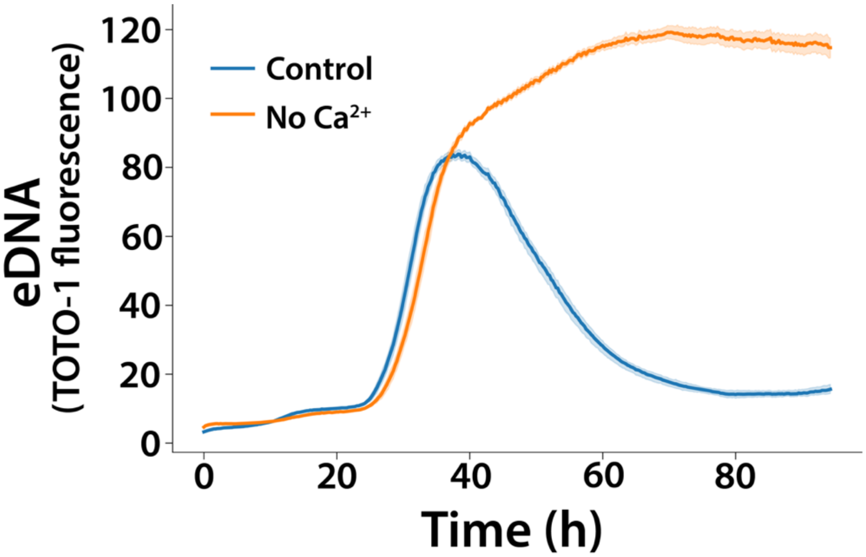

Supplementary Figure 7. eDNA degradation and nuclease activity is lost in the absence of Ca^{2+} in pellicle biofilms. eDNA dynamic of wildtype pellicle biofilms in normal MSgg over development compared to wildtype pellicle biofilms in MSgg without Ca^{2+} added (n=3). Traces are the mean and shaded area is the standard deviation.

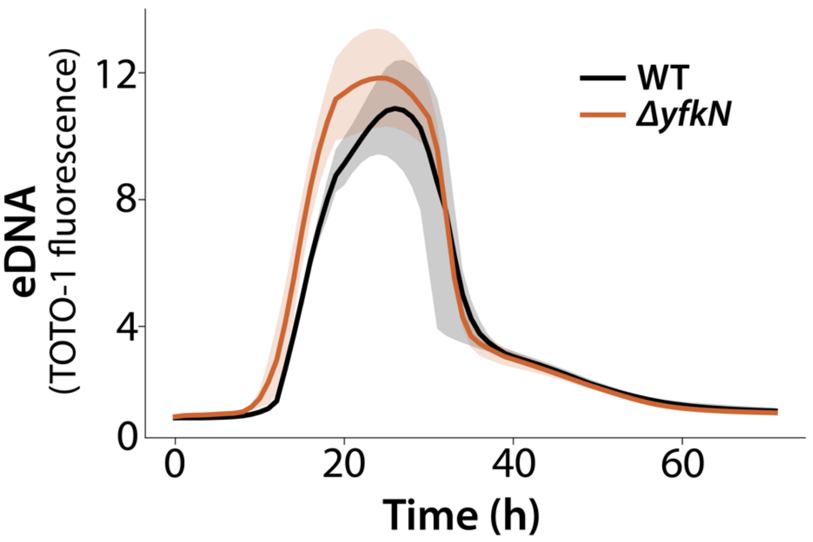

Supplementary Figure 8. *ΔyfkN* mutant biofilms have eDNA pulse including eDNA degradation. eDNA dynamics of wildtype and *ΔyfkN* mutant biofilms on solid MSgg medium. Traces are mean fluorescence of biofilms (n=3), and shaded area is standard deviation for all biofilms.

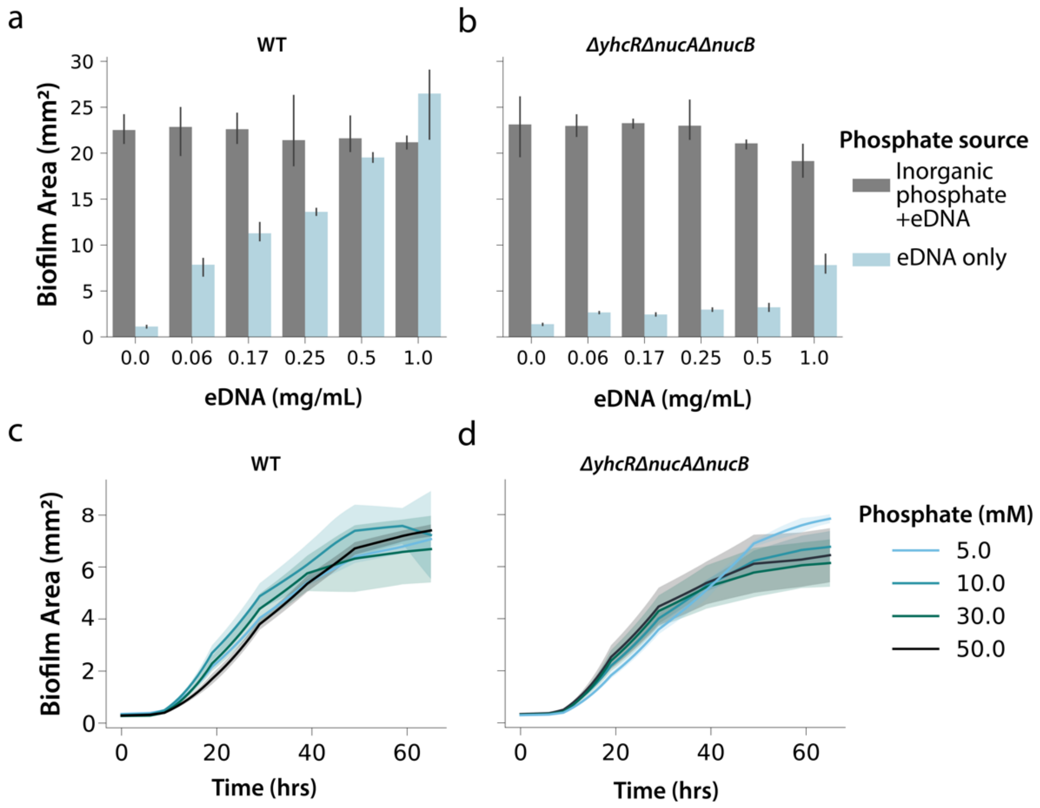

Supplementary Figure 9. Three nucleases, YhcR, NucA, and NucB reclaim exogenous eDNA in colony biofilms to increase biofilm fitness, and do not have altered growth rates with excess phosphate. Area of scanned biofilms grown on MSgg for 66 hours with varying levels of inorganic phosphate with exogenous eDNA or only eDNA as the phosphate source **a,** WT and **b,** *ΔyhcRΔnucAΔnucB* (n=3). Error bars represent the standard deviation. Area of **c,** WT and **d,** *ΔyhcRΔnucAΔnucB* biofilms grown with excess phosphate provided in MSgg for 66 hours (n=3). Error bars represent the standard deviation.

Supplementary Movie 1. eDNA pulse during *B. subtilis* biofilm development. eDNA dynamics were measured using TOTO-1 fluorescent extracellular nucleic acid binding dye over the development of a wildtype *B. subtilis* NCIB 3610 colony biofilm.
